# Gag-Protease Sequence Evolution Following Protease Inhibitor Monotherapy Treatment Failure in HIV-1 Viruses Circulating in East Africa

**DOI:** 10.1089/aid.2015.0138

**Published:** 2015-10-01

**Authors:** Katherine A. Sutherland, Ruth L. Goodall, Adele McCormick, Anne Kapaata, Fred Lyagoba, Pontiano Kaleebu, Geant Thiltgen, Charles F. Gilks, Moira Spyer, Cissy Kityo, Deenan Pillay, David Dunn, Ravindra K. Gupta

**Affiliations:** ^1^ University College London, London, United Kingdom.; ^2^ MRC Clinical Trials Unit at University College London, London, United Kingdom.; ^3^ Uganda Research Unit on AIDS, Medical Research Council (MRC), Uganda Virus Research Institute, Entebbe, Uganda.; ^4^ School of Population Health, University of Queensland, Brisbane, Australia.; ^5^ Joint Clinical Research Centre, Kampala, Uganda.; ^6^ Wellcome Trust Africa Centre for Health and Population Sciences, University of KwaZulu Natal, Mtubatuba, South Africa.

## Abstract

Around 2.5 million HIV-infected individuals failing first-line therapy qualify for boosted protease inhibitor (bPI)-based second-line therapy globally. Major resistance mutations are rarely present at treatment failure in patients receiving bPI and the determinants of failure in these patients remain unknown. There is evidence that Gag can impact PI susceptibility. Here, we have sequenced Gag-Protease before and following failure in 23 patients in the SARA trial infected with subtypes A, C, and D viruses. Before bPI, significant variation in Protease and Gag was observed at positions previously associated with PI exposure and resistance including Gag mutations L449P, S451N, and L453P and Protease K20I and L63P. Following PI failure, previously described mutations in Protease and Gag were observed, including those at the cleavage sites such as R361K and P453L. However, the emergence of clear genetic determinants of therapy failure across patients was not observed. Larger Gag sequence datasets will be required to comprehensively identify mutational correlates of bPI failure across subtypes.

The global scale up of antiretroviral (ART) therapy in resource-limited settings has reached an estimated 12.9 million HIV-infected individuals.^1^ Most patients initiate ART on nucleoside reverse transcriptase inhibitor (NRTI) and nonnucleoside reverse transcriptase inhibitor (NNRTI)-containing regimens, with the protease inhibitor (PI) drug class reserved for use in combination second-line therapies, as recommended by the WHO.^2^ Given the expense and toxicity of PI-containing combination regimens, clinical trials have explored a number of simplification strategies.

The Boosted Protease Inhibitor Monotherapy as Maintenance Second-Line Antiretroviral Therapy in Africa (SARA) trial was a nested pilot study within the DART trial^3^ designed to test whether boosted lopinavir (LPV/r) monotherapy (bPImono) after an initial 24 weeks on second-line combination therapy resulted in similar outcomes to continuation on combination second-line therapy (CT).^4^ The trial demonstrated noninferiority of LPV/r monotherapy in CD4^+^ T cell response and rate of serious adverse events (SAEs), but viremia (≥50 copies/ml) was more common 24 weeks after randomization in the bPImono arm (23% CT vs. 40% bPImono, *p* = 0.01). Major resistance mutations in protease were detected in 5/20 (25%) bPImono participants with a viral load (VL) >1,000 copies/ml at 24 weeks/last time point with successful genotyping (compared to 0/8 CT).^4^ However, the causes of treatment failure in the remaining patients without major drug resistance mutations are unknown.

Studies have shown that mutations in the protease enzyme substrate Gag can also confer PI resistance, with mutations identified in the cleavage sites and within the Gag polyprotein subunits.^5^ Full-length Gag is not included in diagnostic genotypic resistance assays, but in research settings we have shown that the inclusion of Gag alongside its coevolved Protease leads to susceptibility levels different from those measured using protease alone.^6,7^ The genetic diversity observed in Gag and Protease could result in polymorphisms overlapping PI resistance mutations in PI-naive, non-B subtype viruses and so could affect treatment outcome.^8,9^

Gag cleavage site mutations pretherapy were associated with virological outcome in MONARK, a trial examining the efficacy of LPV/r monotherapy as first-line treatment.^10^ MONARK enrolled patients infected mainly with subtype B and CRF02_AG viruses predominant in Europe/North America and West Africa, respectively. PI-naive subtype CRF02_AG viruses from MONARK demonstrated reduced PI susceptibility, hence we hypothesize that the presence of Gag mutations as polymorphisms in PI-naive non-B subtypes may predispose patients to treatment failure on PI monotherapy.^8^ Given the extensive sequence variation in Gag between HIV-1 non-B subtypes, it is necessary to examine the role of Gag cleavage site mutations in PI failure in viruses of other subtypes. We set out to investigate the role of Gag cleavage site and noncleavage site mutations both present pretreatment and evolving during PI exposure, as well as protease resistance mutations in PI treatment failure in subtypes A, C, and D-infected patients enrolled in the SARA trial.

Twenty-eight patients experienced treatment failure in the SARA trial with successful protease genotyping at failure. We amplified by PCR and sequenced *gag* and *protease* genes by population sequencing from pre-PI and treatment failure time point samples for each patient. This study included 23 patients from whom full-length *gag* was successfully amplified at both time points: 16 patients randomized to the boosted PI monotherapy (bPImono) arm and 7 to the continuation therapy arm (CT)^4^ (accession numbers KT351803–KT351848). The frequency of mutations of interest in PI-naive viruses was examined and pairwise comparison performed to determine any change in mutation frequency at the time of treatment failure. Protease sequences were compared to the IAS list of resistance mutations,^11^ Gag cleavage sites were compared to the HIV-1 group M consensus sequence derived from GenBank,^9^ and Gag noncleavage site mutations were examined by comparison to a list of previously described mutations.^5^ Mutation frequency by subtype was determined using the Los Alamos HIV database AnalyzeAlign tool (www.hiv.lanl.gov/content/sequence/QUICK_ALIGNv2).

Pretherapy protease sequences were aligned and compared to the IAS list of resistance mutations.^11^ None of the patients harbored major PI resistance mutations at baseline, although 22/23 had minor PI resistance mutation(s) at baseline at the following protease positions, some of which represent consensus residues in specific non-B subtypes: 10, 16, 20, 33, 36, 60, 63, 69, 77, 89, and 93 (Supplementary Figure S1; Supplementary Data are available online at www.liebertpub.com/aid). Of note, mutations M36I and H69K were present in all subtype A and C patient viruses and I93L was present in all subtype C viruses, and were consensus residues in each subtype, respectively. Other common mutations included L10V (observed in four patients), G16E (four patients), and K20R (six patients), none of which was a consensus residue in subtype A, C, or D. Of particular relevance to this study were mutations associated with resistance to boosted lopinavir: L10I (three subtype A patients), L10V (one subtype A), K20R (five subtype As and 1 subtype C), L33V (one subtype D), L63P (one subtype A, two subtype Cs, and three subtype Ds).

The presence of minor resistance mutations in protease in non-B subtypes has been widely reported: 63P is the consensus residue in subtype D, L10I is present in 19% of subtype A sequences, and K20R is present in 22% of subtype A and 15% of subtype Cs. Previous studies have shown that they do not appear to affect *in vitro* susceptibility; however, these studies have been performed using *in vitro* assays that do not include patient-derived coevolved Gag, which can substantially impact susceptibility.^6,7,12^

Pretherapy Gag cleavage site sequences for each patient were manually compared with the consensus group M sequence to examine the frequency of previously described mutations in these non-B subtypes that could predispose patients to treatment failure (Tables 1, 2, and 3). The p2/NC cleavage site was the most variable as described previously,^10^ with all patients exhibiting at least one mutation in comparison with consensus M in p2/NC. Variability was maintained when comparison within subtype consensus at p2/NC was performed, with 11/11 subtype A, 5/7 subtype C, and 3/5 subtype D patients demonstrating at least one mutation in comparison with their respective subtype consensus sequences at this cleavage site. Excluding the p2/NC cleavage site, 18 of 23 patients had cleavage site mutations versus consensus M at baseline (11/11 subtype A patients, 3/5 subtype D patients, and 4/7 subtype C patients) and 15 versus their respective subtype consensus sequence (8/11 subtype A, 3/5 subtype D, and 4/7 subtype C). Four patients exhibited mutations in the Gag N terminal cleavage sites, which were not subtype consensus residues: two in MA/CA and two in CA/p2. At the C terminus, 17/23 patients had amino acid changes versus consensus M including mutations L449P, S451N, and P453L that previously were associated with PI exposure and/or resistance.^5^

**Table 1.** Variation in Gag Cleavage Sites in Subtype A Viruses

			*Gag Cleavage Sites*				
*Patient number*	*Trial arm*	*Time point*	*MA/CA 128–137 VSQNY/PIVQN*	*CA/p2 359–368 KARVL/AEAMS*	*P2/NC 373–382 TXXIM/MQRGN*	*NC/p1 428–437 ERQAN/FLGKI*	*p1/p6 444–453 RPGNF/LQSRP*
1	CT	Pre-PI	-----/-----	-----/-----	HTH--/-----	^EK^----/-----	-----/P----
		Failure	-----/-----	--K--/-----	HTN--/-----	-----/-----	-----/P----
2	bPImono	Pre-PI	-----/-----	-----/-----	QTS--/--K-M	-----/-----	-----/P----
		Failure	-----/-----	-----/-----	HTN--/--K-M	-----/-----	-----/P----
3	bPImono	Pre-PI	-----/-----	---I-/-----	QPN--/-----	-----/-----	-----/P-N-L
		Failure	-----/-----	--KI-/-----	Q-N--/-----	-----/-----	-----/P-N-L
4	bPImono	Pre-PI	-----/-----	-----/-----	QTN--/--^RK^--	-----/-----	-----/P---^PL^
		Failure	-----/-----	-----/-----	QTN--/--K--	-----/-----	-----/P----
5	bPImono	Pre-PI	-----/-----	-----/-----	^NHAT^---/-----	-----/-----	-----/P----
		Failure	-----/-----	-----/-----	H-N--/-----	-----/-----	-----/P----
6	bPImono	Pre-PI	-----/-----	-----/-----	NTK--/-----	-----/-----	-----/P---L
		Failure	-----/-----	-----/-----	NTK--/-----	-----/-----	-----/P---L
7	bPImono	Pre-PI	+----/-V---	-----/-----	NTK--/-----	-----/-----	-----/P---L
		Failure	-----/-V---	-----/-----	QPN--/-----	-----/-----	-----/P--^RK^L
8	bPImono	Pre-PI	-----/-----	-----/----K	PTN--/I----	-----/-----	-----/P----
		Failure	-----/-----	-----/----K	PTN--/I----	-----/-----	-----/P---L
9	bPImono	Pre-PI	-----/-----	-----/-----	QTNV-/-----	-----/-----	-----/P----
		Failure	-----/-----	-----/-----	QTSV-/-----	-----/---^RK^-	-----/P----
10	bPImono	Pre-PI	-----/-----	-----/-----	NTN--/-----	-----/---RL	-----/P----
		Failure	-----/-----	-----/-----	NTN--/-----	-----/---RL	-----/P----
11	CT	Pre-PI	-----/-----	-----/-----	PTN--/-----	-----/-----	-----/P----
		Failure	-----/-----	-----/-----	PTN--/-----	-----/-----	-----/P----

The sequence at each of the Gag cleavage sites is shown for the 11 patients infected with subtype A viruses, both at pre-PI therapy and at treatment failure time points. Sequences were compared with the HIV-1 group M consensus sequence^9^ using HXB2 numbering. Where no consensus residue was derived an X is present. Deletions are shown by + and mixed residues by superscript letters. Clinical trial arm is shown: boosted PI monotherapy (bPImono) and continuation on combination therapy (CT).

**Table 2.** Variation in Gag Cleavage Sites in Subtype C Viruses

			*Gag cleavage sites*				
*Patient number*	*Trial arm*	*Trial week failure (pre-PI)*	*MA/CA 128–137 VSQNY/PIVQN*	*CA/p2 359–368 KARVL/AEAMS*	*P2/NC 373–382 TXXIM/MQRGN*	*NC/p1 428–437 ERQAN/FLGKI*	*p1/p6 444–453 RPGNF/LQSRP*
12	CT	Pre-PI	-----/-----	-----/-----	N-N--/-----	-----/-----	-----/-----
		Failure	G----/-----	-----/-----	NTN--/-----	-----/-----	-----/-----
13	CT	Pre-PI	-----/-----	-----/-----	N-NV-/-----	-----/-----	-----/--N--
		Failure	-----/-----	-----/-----	N-NV-/-----	-----/-----	-----/--N--
14	CT	Pre-PI	-----/-----	-----/-----	H-A--/--KS-	-----/-----	-----/--N--
		Failure	-----/-----	-----/-----	NTN--/--KS-	-----/-----	-----/--N--
15	bPImono	Pre-PI	I----/-----	-----/-----	NSN-L/---S-	-----/-----	-----/--N--
		Failure	-----/-----	-----/-----	NSN-L/---S-	---V-/-----	-----/--N--
16	CT	Pre-PI	-----/-----	-----/-----	NSN--/-----	-----/-----	-----/-----
		Failure	-----/-----	-----/-----	NSN--/-----	-----/-----	-----/-----
17	bPImono	Pre-PI	-----/-----	-----/-----	NSN--/----^NK^	-----/-----	-----/--N--
		Failure	-----/-----	-----/-----	NSN--/-----	-----/-----	-----/--N-L
18	bPImono	Pre-PI	-----/-----	-----/-----	NN^NH^--/---S-	-----/-----	-----/-----
		Failure	-----/-----	-----/-----	NNH--/---S-	-----/-----	-----/-----

The sequence at each of the Gag cleavage sites is shown for the seven patients infected with subtype C viruses, both at pre-PI therapy and at treatment failure time points. Sequences were compared with the HIV-1 group M consensus sequence^9^ using HXB2 numbering. Where no consensus residue was derived an X is present and mixed residues are shown in superscript letters. Clinical trial arm is shown: boosted PI monotherapy (bPImono) and continuation on combination therapy (CT).

**Table 3.** Variation in Gag Cleavage Sites in Subtype D Viruses

			*Gag cleavage sites*				
*Patient number*	*Trial arm*	*Trial week failure (pre-PI)*	*MA/CA 128–137 VSQNY/PIVQN*	*CA/p2 359–368 KARVL/AEAMS*	*P2/NC 373–382 TXXIM/MQRGN*	*NC/p1 428–437 ERQAN/FLGKI*	*p1/p6 444–453 RPGNF/LQSRP*
19	CT	Pre-PI	-----/-----	-----/-----	QPN--/-----	^ED^----/----^VL^	-----/-----
		Failure	-----/-----	-----/-----	QSN--/-----	-----/----V	-----/-----
20	bPImono	Pre-PI	-----/-----	-----/-----	N-A--/-----	-----/-----	-----/-----
		Failure	-----/-----	-----/-----	N-A--/-----	-----/-----	-----/-----
21	bPImono	Pre-PI	-----/-----	-----/-----	^SNTA^--/-----	-----/-----	-----/-----
		Failure	-----/-----	-----/-----	-TA--/-----	-----/-----	-----/-----
22	bPImono	Pre-PI	-----/-----	-----/-----	NTA--/-----	-----/-----	-----/--N--
		Failure	-----/-----	-----/-----	N^AT^A--/-----	-----/-----	-----/--N--
23	bPImono	Pre-PI	-----/-----	-----/-----	N-A--/-----	-----/-----	-----/--N--
		Failure	-----/-----	-----/-----	N-A--/-----	-----/-----	-----/--N--

The sequence at each of the Gag cleavage sites is shown for the five patients infected with subtype D viruses, both at pre-PI therapy and at treatment failure time points. Sequences were compared with the HIV-1 group M consensus sequence^9^ using HXB2 numbering. Where no consensus residue was derived an X is present and mixed residues are shown in superscript letters. Clinical trial arm is shown: boosted PI monotherapy (bPImono) and continuation on combination therapy (CT).

The L449P mutation was present in all patients infected with subtype A (*n* = 11), in keeping with the finding of another study reporting P as the consensus amino acid at position 449 in subtype A.^9^ The cleavage site mutation S451N was found in 7/23 patients (4/7 subtype C, 2/5 subtype D, and 1/11 subtype A), but is not a consensus residue in these subtypes. Mutation P453L was present in 4/11 subtype A patients, but again is not a consensus residue in subtypes A, C, or D. Only 5/23 patients had no cleavage site mutations in comparison with consensus M outside of the p2/NC site (3/7 subtype C and 2/5 subtype D).

Multiple amino acid changes in Gag outside cleavage sites have been reported to affect susceptibility to PIs and to be associated with treatment failure. Therefore we manually examined the sequences for the presence of these previously described mutations in the pre-PI sequences.^5^ Several of these mutations were present pre-PI therapy in our patient cohort, at positions 12, 30, 62, 76, 79, 81, 371, 389, 401, and 456. E12K, described as accelerating the emergence of resistance in concert with other Gag mutations, was the most frequent, present in 21/23 patients, and is in fact the consensus amino acid in most non-B subtypes.^13^ Mutations in the matrix (MA) subunit described by Parry *et al*. were also common with R76K in 13/23 patients (6/11 subtype A, 4/7 subtype C, and 3/5 subtype D) and Y79F in 10/23 patients (3/11 subtype A, 4/7 subtype C, and 3/5 subtype D); in fact, 76K is the consensus residue in subtype D viruses.^14^

Pairwise comparisons of pre-PI and failure Gag-Protease sequences from 23 patients were performed to identify amino acid changes associated with PI exposure and treatment failure. Changes in protease at failure in comparison with the pre-PI sample were found in 17/23 patients (Table 4). Excluding the development or fixation of a mixed amino acid position, 12/23 patients had a new mutation at treatment failure. The major LPV resistance mutation I54V developed at failure in two patients (6 and 8) and V82A in two additional patients (4 and 15). Two patients developed a new minor resistance mutation at failure (patient 3, K20R and 4, L10I) and two patients had mutations at minor resistance position 63, though to different residues (16 to I and 11 to S).^11^

**Table 4.** Mutations in Protease at Time of Treatment Failure in Comparison with the Pre-Protease Inhibitor Time Point

		*Protease mutations by amino acid position (pre-PI residue—failure residue)*																											
*Patient*	*Subtype*	*10*	*12*	*13*	*14*	*15*	*16*	*19*	*20*	*24*	*33*	*36*	*37*	*39*	*43*	*46*	*50*	*54*	*57*	*63*	*64*	*66*	*69*	*70*	*72*	*74*	*77*	*82*	*89*
1	A																												
2	A	V-L			K-K/R		E-G		R-K					S-P					R-K					K/R-K					
3	A				R-K			L-L/S	K-R/T/K		L-L/S				R-R/K											T-A			
4	A	L-I														M-M/I			R-K								V/I-I	V-V/A	
5	A																												
6	A																	I-V											
7	A	V/L-L								L/S-L																			
8	A																	I-V	R-K	P-L									
9	A																						R-K/R						
10	A																												
11	A						E-G																			V-M			
12	C								R-K																				L-M
13	C																					M- I							
14	C																												
15	C																											V-A	
16	C		T-S										D-N				P-I				L-I				V-I				L-F
17	C																						K-R						
18	C																												
19	D			V-V/I																									
20	D			V-V/I		I/V-I																							
21	D																												
22	D											M-I/M															I-V/I		
23	D					I/V-I																			V-I				

Mutations are shown in the following format: pre-PI residue, failure residue. Where mixed residues were present, they are shown by ‘/’.

At the time of treatment failure 16 patients had Gag cleavage site changes in comparison with baseline (excluding fixation of mixed residues) at positions 128, 361, 373, 374, 375, 431, and 453 (Tables 2, 3, and 4). Of these, 13 patients displayed a new change and 10 displayed a reversion to consensus M amino acid; seven patients had both a new mutation and a reversion. The p2/NC cleavage site displayed the greatest variation between baseline and failure, with 12 patients displaying at least one amino acid change after PI therapy (mostly at positions 373, 374, and 375). In patient 15, the A431V mutation developed in the NC/p1 cleavage site at failure with major mutation V82A, a coevolution that is frequently observed.^5^ Of particular interest, two CSMs developed in two separate patients at the time of failure—R361K (in two subtype A patients—1 and 3) and P453L (in subtype A patient 8 and subtype C patient 17). To our knowledge R361K has not been previously linked to PI resistance or exposure, but P453L has been associated with PI exposure *in vitro*, *in vivo*, and with PI resistance.^5^

Pairwise comparisons showed that 16 of 23 patients had at least one amino acid change at previously described noncleavage site residues at treatment failure in comparison with pre-PI. These changes occurred at the following positions in Gag: 30 (*n* = 6 patients), 62 (*n* = 6), 75 (*n* = 2), 76 (*n* = 5), 79 (*n* = 4), 81 (*n* = 1), 112 (*n* = 1), 370 (*n* = 4), 371 (*n* = 1), 389 (*n* = 2), 390 (*n* = 1), 401 (*n* = 3), 456 (*n* = 1), and 468 (*n* = 4). However, in general no consistent pattern of selection toward specific amino acids at each position was observed across a number of patients. For example, the R76K mutation has previously been described as affecting PI susceptibility but here selection from an R/K mix pre-PI to each of Q and R at failure was observed in different patients, as well as R76K, K76R/K, and K76R in three other patients.^14^ The development of Y79F at failure was present in two patients, although F79Y was also observed in a third, and T81A was also observed at failure in a single patient. Mutations at positions 76, 79, and 81 were previously described in concert, but in this patient cohort they were not observed in the same patients.^14^

We have demonstrated variation at baseline in Gag-Protease of non-B viruses at sites of interest for PI resistance and evolution at these sites during PI therapy. However, the emergence of clear genetic determinants of therapy failure across a number of patients was not observed. This may be due to the limited number of patients available for each viral subtype, as the intersubtype differences confound the search for resistance mutations associated with treatment failure. Another possibility is that given the large number of CTL epitopes present in Gag, the HLA type of the patient would affect the development of resistance mutations in Gag. We also cannot rule out the role of other regions outside of Gag and Protease in PI resistance, such as envelope,^15^ and the potential role of unreported poor therapy adherence in treatment failure. There is an urgent need for a greater understanding of PI therapy failure as these drugs become more widely used in populations infected with divergent HIV-1 strains and further genotypic and phenotypic studies examining determinants of PI treatment failure in non-B subtypes are required.

## Supplementary Material

Supplemental data
