# Supplementary material for: Gag-Protease Sequence Evolution Following Protease Inhibitor Monotherapy Treatment Failure in HIV-1 Viruses Circulating in East Africa
Source: AIDS Res Hum Retroviruses. 2015 Oct 1;31(10):1032–7. doi: 10.1089/aid.2015.0138 (PMC4675176; doi:10.1089/aid.2015.0138)
Supplement: Supplemental data [file Supp_Data.pdf]

Supplementary Data

```
1(A)   PQITLWQRPLVTVKVGGQLREALLDTGADDTVLEDINLPGKWKPKMIGGIGGFIKVKQYDQILIEICGKKAIGTVLVGPTPVNIIGRNMLTQIGCTLNF
2(A)   PQITLWQRPVVTVKIEGQLREALLDTGADDTVLEDINLSGKWKPKMIGGIGGFIKVRQYDQILIEICGK\AIGTVLVGPTPVNIIGRNMLTQIGCTLNF
3(A)   PQITLWQRPLVTVRVGGQLKEALLDTGADDTVLEDIDLPGKWRPKMIGGIGGFIKVKQYDQILIEICGKKAIGTVLVGPTPVNIIGRNMLTQIGCTLNF
4(A)   PQITLWQRPLVTV\IGGQLIEALLDTGADDTVLENINLPGKWKPKMIGGIGGFIKVRQYDQILIEICGKKAIGTVL/GPTPVNIIGRNMLTQIGCTLNF
5(A)   PQITLWQRPLVTVKIGGQLKEALLDTGADDTVLEDINLPGKWKPKMIGGIGGFIKVKQYEQILIEICGKKAIGTVLVGPTPVNIIGRNMLTQIGCTLNF
6(A)   PQITLWQRPLVTVKIGGQLKEALLDTGADDTVLEDINLPGKWKPKMIGGIGGFIKVKQYDQILIEICGKKAIGTVLVGPTPVNIIGRNMLTQIGCTLNF
7(A)   PQITLWQRP+VTVKIGGQLKEAL%DTGADDTVLEDINLPGKWKPKMIGGIGGFIKVRQYDQILIEICGKKAIGTVLVGPTPVNIIGRNMLTQIGCTLNF
8(A)   PQITLWQRPLVTVKIGGQLKEALLDTGADDTVLEDINLPGKWKPKMIGGIGGFIKVRQYDQIPIEICGKKAIGTVLVGPTPVNIIGRNMLTQIGCTLNF
9(A)   PQITLWQRPLVTVKIGGQLIEALLDTGADDTVLEDINLPGKWKPKMIGGIGGFIKVRQYDQILIEICGRKAIGTVLVGPTPVNIIGRNMLTQIGCTLNF
10(A)  PQITLWQRPIVTIKIGGQLKEALLDTGADDTVLEDINLPGKWKPKMIGGIGGFIKVKQYDEILIEIYGKKAIGTVLVGPTPVNIIGRNMLTQIGCTLNF
11(A)  PQITLWQRPVVTVKIEGQLKEALLDTGADDTVLEDINLPGKWKPKMIGGIGGFIKVKQYEQILIEICGKKAIGTVLVGPTPVNIIGRNMLTQIGCTLNF
12(C)  PQITLWQRPLVSIKVGGQVREALLDTGADDTVLEEIKLPGNWKPKMIGGIGGFIKVRQYDQILIEICGKKAIGTVLVGPTPVNIIGRNLLTQLGCTLNF
13(C)  PQITLWQRPLVTIKVGGQIKEALLDTGADDTVLEDIELPGRWKPKMIGGIGGFIKVRQYDQIPIEICGKKAIGTVLVGPTPVNIIGRNLLTQLGCTLNF
14(C)  PQITLWQRPLVPIKVEGQIKEALLDTGADDTVLEEINLPGRWKPKMIGGIGGFIKVRQYDQITIEICGKKAVGTVLVGPTPVNIIGRNLLTQLGCTLNF
15(C)  PQITLWQRPLVSIKVGGQIKEALLDTGADDTVLEEINLPGKWKPKMIGGIGGFIKVRQYDQIPIEICGKKAIGTVLVGPTPVNIIGRNLLTQLGCTLNF
16(C)  PQITLWQRPLVTIKVGGQIKEALLDTGADDTVLEEIDLPGKWKPKMIGGIGGFIKVRQYEEIPLEICGKKAVGTVLVGPTPVNIIGRNLLTQLGCTLNF
17(C)  PQITLWQRPLVSIKVGGQIKEALLDTGADDTVLEEINLPGKWKPKMIGGIGGFIKVRQYDQIVIEICGKKAIGSVLVGPTPVNIIGRNMLTQLGCTLNF
18(C)  PQITLWQRPLVSIKIGGQIKEALLDTGADDTVLEEINLPGKWKPKMIGGIGGFIKVRQYDQITIEICGKKAIGSVLVGPTPVNIIGRNMLTQLGCTLNF
19(D)  PQITLWQRPLVTVKIGGQLKEALLDTGADDTVVEEMNLPGKWKPKMIGGIGGFIKVKQYDEIPIDICGHKAIGTVLVGPTPVNIIGRNLLTQIGCTLNF
20(D)  PQITLWQRPLVTVK/GGQLKEALLDTGADDTVLEEINLPGKWKPKMIGGIGGFIKVRQYDQIPIEICGHKAIGTVLVGPTPVNIIGRNLLTQIGCTLNF
21(D)  PQITLWQRPLVPIKVEGQLKEALLDTGADDTVLEDINLPGRWKPKMIGGIGGFIKVRQYDQITIEICGHKATGTVLVGPTPVNIIGRNMLTQIGCTLNF
22(D)  PQITLWQRPLVTVKIGGQLKEALLDTGADDTVLEDMNLPGKWKPKMIGGIGGFIKVKQYDQIPIEICGYKAVGTVLIGPTPVNIIGRNLLTQIGCTLNF
23(D)  PQITLWQRPLVTIKIGGQLKEALLDTGADDTVLEEINLPGKWKPKMIGGIGGFIKVRQYDQILVEICGHKAVGTVLVGPTPVNIIGRNLLTQIGCTLNF
```

SUPPLEMENTARY FIG. S1. Pre-protease inhibitor (PI) amino acid sequences for all patients. The viral subtype is shown in brackets after the patient number. Mixed amino acids at a position are depicted by the following symbols: K/R (\), I/V (/), V/L (+), and L/S (%).
